# The Rise and Fall of Chloroquine/Hydroxychloroquine as Compassionate Therapy of COVID-19

**DOI:** 10.3389/fphar.2021.584940

**Published:** 2021-05-06

**Authors:** Elangovan Manivannan, Chandrabose Karthikeyan, N. S. Hari Narayana Moorthy, Subash Chandra Chaturvedi

**Affiliations:** ^1^ School of Pharmacy, Devi Ahilya Vishwavidyalaya, Indore, India; ^2^ Department of Pharmacy, Indira Gandhi National Tribal University, Amarkantak, India; ^3^ Department of Pharmacy, Shri Aurobindo Institute of Medical Sciences, Indore, India

**Keywords:** chloroquine, hydroxychloroquine, coronavirus, COVID-19, SARS-CoV-2

## Abstract

The emergence and rapid spread of novel coronavirus disease (COVID-19) has posed a serious challenge to global public health in 2020. The speed of this viral spread together with the high mortality rate has caused an unprecedented public health crisis. With no antivirals or vaccines available for the treatment of COVID-19, the medical community is presently exploring repositioning of clinically approved drugs for COVID-19. Chloroquine (CQ) and hydroxychloroquine (HCQ) have emerged as potential candidates for repositioning as anti–COVID-19 therapeutics and have received FDA authorization for compassionate use in COVID-19 patients. On March 28, 2020, the U.S. Food and Drug Administration (FDA) issued an Emergency Use Authorization (EUA) for HCQ in the treatment of COVID-19. However, it was later revoked by the FDA on June 15, 2020, after analyzing the emerging scientific data from ongoing clinical trials. Similarly, the World Health Organization (WHO) also conducted a Solidarity trial of chloroquine, hydroxychloroquine, remdesivir, lopinavir, and ritonavir. However, on May 23, 2020, the executive body of the “Solidarity trial” decided to put a temporary hold on the HCQ trial. On June 17, 2020, the WHO abruptly stopped the Solidarity trial of HCQ. The current review strives to examine the basis of compassionate use of CQ and HCQ for the treatment of COVID-19 in terms of literature evidence, establishing the antiviral efficacy of these drugs against corona and related viruses. Furthermore, the review presents a critical analysis of the clinical trial findings and also provides an insight into the dynamically changing decision on the authorization and withdrawal of HCQ as anti–COVID-19 therapy by the U.S. FDA and the WHO. Ultimately, our study necessitates an evidenced-based treatment protocol to confront the ongoing COVID-19 pandemic and not the mere observational study that mislead the public healthcare system, which paralyzes the entire world.

## Introduction

An emergence of a mysterious viral disease causing a cluster of unexplained pneumonia and bronchiolitis cases was first registered in Wuhan, Hubei Province, China (Huang C. et al., 2020). This severe acute respiratory (SAR) disease was recognized in late December 2019 and reportedly given the name coronavirus disease, 2019 (COVID-19) [coronavirus disease (COVID-2019) technical guidance, WHO]. The causative agent for the disease was identified to be a novel coronavirus (SARS-CoV-2) (Wu et al., 2020). In a short period, COVID-19 spread rapidly and progressed to an epidemic proportion throughout the world with substantial morbidity and mortality. Therefore, COVID-19 has been declared a major public health emergency by the World Health Organization (WHO). On March 11, 2020, the WHO confirmed the COVID-19 outbreak as a global pandemic (Cucinotta and Vanelli, 2020). As of March 29, 2021, COVID-19 is responsible for more than 126,890,643 infections and 2,778,619 deaths worldwide (novel coronavirus 2019 status report, WHO). In India, there are 12,039,644 confirmed COVID-19 cases, and 161,843 deaths have been recorded as of March 29, according to the COVID-19 web portal, Ministry of Health and Family Welfare, Govt. of India.

Since the outbreak of the COVID-19 pandemic, tremendous efforts have been invested in the development of vaccines and antiviral therapeutics that target SARS-CoV-2 (Amanat and Krammer, 2020). At present, there is no Food and Drug Administration (FDA)-approved specific drug available for the treatment of COVID-19. Therefore, infection control measures like quarantine, isolation, social distancing, and travel ban are strictly imposed worldwide to contain the disease [Institute of Medicine (US) Forum on Microbial Threats, 2007]. COVID-19 patients are given supportive care such as fluid support, oxygen, and ventilatory support. The severe cases of COVID-19 are given mechanical ventilation or extracorporeal membrane oxygenation (ECMO) as life support (Nakamura et al., 2020). Besides this, several FDA-approved drugs have been repurposed based on preliminary clinical findings for the treatment of COVID-19 patients on a compassionate basis. The putative treatment based on the concept of drug repurposing includes chloroquine (CQ) (Shukla et al., 2020), hydroxychloroquine (HCQ) (Shukla et al., 2020), lopinavir (Chu et al., 2004), ritonavir (Cao et al., 2020), remdesivir (Wang et al., 2020), ribavirin (Khalili et al., 2020), griffithsin (Li and Clercq, 2020), tocilizumab (Marotto and Sarzi-Puttini 2020), sarilumab (Sallard et al., 2020), interferon (Long et al., 2020), immunoglobulins (Jiang et al., 2020; Long et al., 2020), and corticosteroids (Zha et al., 2020) used to reduce the viral load and prevent lung damage.

This mini-review aimed to provide a summary of the therapeutic potential and experimental use of CQ and HCQ in fighting COVID-19. Herein, we summarize the basis for compassionate use of CQ and HCQ, their *in vitro* and *in vivo* antiviral activities on coronaviruses, and clinical trials on COVID-19 patients. The review presents a critical analysis of the clinical trial findings and also provides an insight into the dynamically changing decision on the authorization and withdrawal of HCQ as anti–COVID-19 therapy by the U.S. FDA and the WHO.

## Compassionate Therapy of COVID-19

### Antiviral Activities of Chloroquine

**FIGURE 1:**
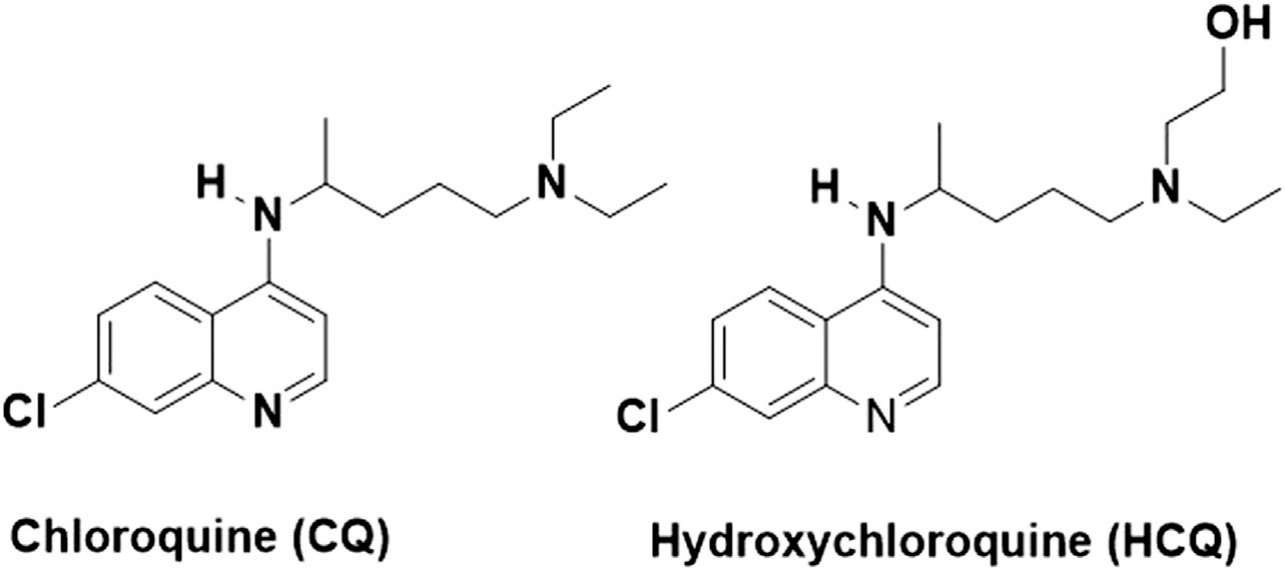
Chemical structures of chloroquine (CQ) and hydroxychloroquine (HCQ).

CQ is chemically represented as N4-(7-chloroquinolin-4-yl)-N1,N1-diethylpentane-1,4-diamine, as shown in Figure 1 (Tse et al., 2019). It is an inexpensive drug that has been used for more than 70 years for the treatment of malaria (Arrow et al., 2004). Although some malaria strains have developed resistance against CQ, it is one of the most widely prescribed drugs for malaria even today. Besides having clinically proven antimalarial activity, CQ demonstrates a wide range of pharmacological activities such as anti-inflammatory, immunomodulatory, and antiviral activities (Browning, 2014).

A large number of publications cite the *in vitro* studies on antiviral properties of CQ against a variety of viruses (Hashem et al., 2020; Huang M et al., 2020). The *in vitro* antiviral effect of CQ was identified for the first time in 1969 (Inglot, 1969), and this is followed by many published reports on antiviral properties of CQ in subsequent years (Shimizu et al., 1972) and in 1981 (Glushakova and Lukashevich, 1989). Further, the anti–SARS-CoV activity (growth inhibition of coronaviruses in cell culture) of both CQ and HCQ was reported in 2005 (Vincent et al., 2005). In addition to antiviral effects, CQ also caused a significant reduction in the expression of pro-inflammatory cytokines, interferons (IFN-β and IFN-g), tumor necrosis factor (TNF-α), and interleukins (IL-6 and IL-12) (Jang et al., 2006). Farias et al. (2014) reported that CQ treatment (dose: 50 mg/ml) resulted in a significantly low virus production in dengue (DENV-2)-infected U937 cells. However, CQ was found to be nontoxic to the normal cells at the same dose (Farias et al., 2014).

Several articles reported the *in vivo* antiviral activity of CQ against human coronavirus OC43 (Keyaerts et al., 2009), enterovirus EV-A71 (Tan et al., 2018), Zika virus (Li et al., 2017), and influenza A H5N1 (Yan et al., 2013). CQ also showed promising *in vitro* antiviral effects on numerous viruses, but the *in vivo* antiviral efficacy of CQ in the primate model of CHIKV infection was not found satisfactory. CQ was found to worsen the disease in the primate model of CHIKV infection by exacerbating the acute fever and delaying the cellular immune response to an incomplete CHIKV viral clearance (Roques et al., 2018). Similarly, CQ was found active *ex vivo* but not *in vivo* in the case of Ebolavirus (Dowall et al., 2015), Nipah virus (Pallister et al., 2009), and influenza virus (Vigerust and McCullers, 2007). CQ has also been tested in chronic hepatitis C and HIV patients for viral clearance. CQ exhibited only a modest level of antiviral effect against chronic hepatitis C infection, and a transient viral load reduction was observed with CQ treatment in a small sample size pilot trial in nonresponder HCV patients (Peymani et al., 2016). However, this was found inadequate for inclusion of the drug in the standardized treatment protocols for hepatitis C–infected patients (Helal et al., 2016). The therapeutic use of CQ in HIV-infected patients has been considered indecisive, and the drug has not been recommended for further use in acquired immune deficiency syndrome (AIDS) treatment (Chauhan and Tikoo, 2015). Overall, CQ exhibited promising *in vitro* antiviral activities against a variety of viruses; however, this preexisting knowledge has not yet been translated into meaningful preclinical studies.

Farias et al. (2014) reported that CQ inhibits the replication of a human coronavirus (HCoV-OC43) in HRT-18 cells, with an effective concentration (EC_50_) of 0.306 ± 0.0091 µm and a lethal concentration (LD_50_) of 419 ± 192.5 µm. The selectivity index observed in HCoV-OC43 was SI. 1,369, which shows the wide safety margin of CQ (Farias et al., 2014). Further, the *in vivo* study of CQ on newborn C57BL/6 mice infected with a lethal HCoV-OC43 infection showed the highest survival rate (98.6%). Overall, the results show the favorable *in vitro* and *in vivo* antiviral effects of CQ against HCoV-OC43 (Farias et al., 2014). Similar *in vitro* antiviral activity studies of CQ on different types of viruses including SARS-CoV-2 were observed in the recent past (Wang et al., 2020). Therefore, it was hypothesized that CQ may be the potential clinical candidate against SARS-CoV-2. As a result of these findings, CQ has already been incorporated into the treatment protocols of certain COVID-19 patients.

### Antiviral Activities of Hydroxychloroquine

HCQ is a hydroxylated derivative of CQ and an effective antimalarial agent (Liu et al., 2020). HCQ is also broadly used for the treatment of various autoimmune diseases, such as rheumatoid arthritis and systemic lupus erythematosus (Silva et al., 2013). HCQ exhibits a better safety profile than CQ, hence is more tolerable than CQ in COVID-19 patients (Gevers et al., 2020). Furthermore, studies have demonstrated that the immunomodulatory activity of HCQ may play an important role in controlling the cytokine storm in severely infected SARS-CoV-2 patients (Silva et al., 2013).

Yao et al. (2020) studied the *in vitro* antiviral properties and rationalized the prophylactic activity of CQ and HCQ. The authors also built physiologically based pharmacokinetic models (PBPKs) for both of these drugs to predict drug concentrations under different dosing regimens. The *in vitro* antiviral activity evaluation of both these drugs was carried out in SARS-CoV-2–infected Vero cells. HCQ (EC_50_ = 0.72 μm) was found to be more active than its counterpart CQ (EC_50_ = 5.47 μm). The EC_50_ values estimated for CQ in Vero cells were 23.90 and 5.47 μm at 24 and 48 h, respectively (Yao et al., 2020). However, the EC_50_ values for HCQ were observed to be 6.14 and 0.72 μm at 24 and 48 h, respectively. It was reported that EC_50_ values for CQ in the drug pretreatment method were >100 and 18.01 μm at 24 and 48 h, respectively. In the same method, EC_50_ values for HCQ were found to be 6.25 and 5.85 μm at 24 and 48 h, respectively. The inhibition rate of CQ did not exceed 50% even at the maximum concentration tested. Both CQ and HCQ were found to decrease viral replication in a concentration-dependent manner. All these results suggested that HCQ demonstrated superior *in vitro* SARS-CoV-2 inhibition in comparison to CQ (Yao et al., 2020).

It was evident from several *in vitro* and *in vivo* antiviral activity studies that HCQ has exhibited potent antiviral activity against coronaviruses (Yao et al., 2020). Furthermore, the drug also elicits tremendous immunomodulatory potential in addition to established clinical safety at appropriate doses (Chandler et al., 2020). All these findings support the inclusion of HCQ in the treatment of COVID-19. However, few reports suggest that even short-term treatment with HCQ can cause cardiac arrhythmias, dermatological reactions, hypoglycemia, and seizures, triggering serious concerns over the use of HCQ in this critical situation (Pereira, 2020). Despite these side effects, both these drugs have been used in the clinical practice of malaria and inflammatory disease for many years. HCQ being a more water soluble and less toxic than CQ is most suitable for repurposing. The promising *in vitro* antiviral activity results of HCQ against SARS-CoV-2 together with better safety profile positioned HCQ as a potential therapeutic option for the treatment of COVID-19.

## Clinical Trials of Chloroquine and Hydroxychloroquine

The high mortality rate and tremendous pressure faced by public health systems to save lives during this devastating COVID-19 pandemic allowed the experimental use of CQ and HCQ in the treatment of severely infected COVID-19 patients (Becker, 2020). Researchers all over the world have initiated clinical trials of the repurposed drugs to find an effective cure for COVID-19. As a result, an enormously large number of clinical trials are underway to generate the robust data needed to establish the therapeutic efficacy and clinical safety of these drugs in COVID-19 cases (Lythgoe and Middleton, 2020). Until December 25, 2020, there were 354 clinical trials that have been registered in various national and international clinical trial databases for CQ and HCQ either alone or in combination with some other drugs in the treatment of COVID-19 (clinicaltrials.gov, WHO).

Last year, Gao et al. (2020) recorded the first clinical trial outcomes of CQ as reported in a news briefing by the Chinese government agency in February 2020. The news briefing revealed that the study was conducted with more than 100 COVID-19 patients. The clinical trial candidate CQ phosphate was found to be much superior to the control treatment in COVID-19. It was also stated that CQ successfully inhibited the exacerbation of pneumonia, improved lung imaging findings, promoted a virus-negative conversion, and shortened the disease course. No specific adverse events were observed in the trial (Gao et al., 2020). It appears that these findings were a result of a compilation of clinical data from several ongoing trials conducted in different Chinese hospitals from a variety of studies. So far, no such clinically validated data are available in the public domain supporting these findings.

A series of studies carried out in the outpatient (COVID-19) setting to test the triple therapy consists of zinc, low-dose hydroxychloroquine, and azithromycin (Derwand et al., 2020). In this retrospective study, a total of 141 COVID-19 patients received triple therapy for 5 days. The results of the study showed that among all treated patients, only 4 were hospitalized and only one patient in the treatment group died compared to 13 patients in the untreated group. Further, the study also reported that no cardiac side effects were observed using triple therapy.

Another pilot study on clinical benefits of HCQ in the treatment of COVID-19 was carried out in China. It was a randomized clinical trial involving 30 confirmed COVID-19 cases. The patients were randomized 1:1 to the HCQ group and the control group. They were given an HCQ plus conventional therapy or conventional treatment alone (Chen J. et al., 2020). On day 7, 13 patients administered with HCQ and 14 of those in the control group were found to be negative for COVID-19 nucleic acid in throat swabs. Other COVID-19 clinical measures like fever (the time required to achieve normal body temperature), progress in pneumonia, and overall clinical improvement were observed similarly in both groups. In contrast to previous studies, few adverse events were reported in the treatment group (Chen Y. et al., 2020). One patient who was given HCQ developed severe lung disease during the course of treatment. This study appears to be an open-label clinical trial with few participants. However, the trial gives no sufficient information on the therapeutic and prophylactic value of HCQ on COVID-19 patients.

Another clinical trial of HCQ was conducted at the University Hospital Institute Méditerranée Infection in Marseille, France. The study included a trial treatment of HCQ and a combination of HCQ with azithromycin in hospitalized COVID-19 patients (Gautret et al., 2020). The trial included a single-arm protocol carried out from early March to March 16th. The patients received 600 mg of HCQ on daily basis, and the viral load was tested every day in their nasopharyngeal swabs (Gautret et al., 2020). Further, azithromycin was added to the regular HCQ treatment depending on the clinical presentation of COVID-19 patients. On day 6 post-inclusion, either the presence or absence of SARS-CoV-2 virus in nasopharyngeal swabs was considered as the endpoint. Among all, twenty COVID-19 patients treated in this study presented a significant reduction of the viral load compared to control groups. It was also observed in the study that a combination of azithromycin and HCQ was more efficient in virus elimination. The overall results of the study showed that all patients treated with HCQ and azithromycin combination were 100% virologically cured as compared to patients (57.1% cured) treated with HCQ alone. A recovery rate of 12.5% was observed in the control group. The major outcome of this clinical trial was that all the patients who were treated with a combination of HCQ and azithromycin tested negative for COVID-19 on day 6 (Gautret et al., 2020). Therefore, the authors advocate the clinical effectiveness of HCQ and a synergistic effect in combination with azithromycin in the treatment of SARS-CoV-2 infection. However, the article received greater attention of scientific community with severe criticism and major concern for the clinical trial results presented in the study (International Society of Antimicrobial Chemotherapy, 2020).

## Use of Hydroxychloroquine in India to Fight COVID-19

In a large and densely populated country like India, the battle against COVID-19 is an enormous challenge. With the onset of the COVID pandemic, India has been facing several issues, such as shortage of diagnostic tools, medical equipment, and related medical supplies. It has directly challenged our public healthcare system and forced to quickly respond. As no drugs were available to fight against the ugly battle of COVID-19, the WHO and the FDA authorized the use of HCQ based on previously available clinical observational studies. Similarly, the Indian Council of Medical Research (ICMR), functioning under the Ministry of Health and Family Welfare, Govt. of India, recommended the use of HCQ in the treatment protocol for COVID-19 patients. India is the major producer and supplier of HCQ, and several countries were helped by additional supplies from India during the pandemic (Batumalai K., 2020; Brian et al., 2020; Channnel News Asia., 2020; NIH Clinical Trials.gov. 2020; Sibbal, 2020; Ying, 2020). The ICMR has also recommended a prophylaxis therapy with HCQ (400 mg twice on day 1, then 400 mg once a week thereafter) for asymptomatic healthcare workers in COVID-19 hospitals and household contacts of confirmed COVID-19 cases (Chatterjee et al., 2020; Rathi et al., 2020; Tilangi et al., 2020). The WHO Solidarity trial is the world's largest global randomized controlled clinical trial, and India contributed one-tenth of the participants in this trial. The authorization to use azithromycin in combination with hydroxychloroquine (HCQ) to treat patients with severe SARS-CoV-2 infections has been rolled back after the interim trial results that showed no potential benefit. During June, 2020, the Ministry of Health and Family Welfare, Govt. of India, issued an updated clinical management protocol for COVID-19 based on the clinical severity of the disease. The revised protocol allows the use of HCQ to COVID-19 patients in the early course of the disease and not on critically ill patients. Furthermore, it was advised to be administered only after “shared decision making with the patients” and also recommends an ECG before prescription. As part of India’s COVID-19 therapeutic management, the Indian government has distributed 111.6 million pills of hydroxychloroquine or HCQ (Government Information in Parliament 2021).

## What Went Wrong With the Hydroxychloroquine?

Some clinical observational studies have suggested therapeutic benefits of HCQ in COVID-19, whereas other studies have shown mixed results. Risch et al. reported that hydroxychloroquine has demonstrated significant major outpatient treatment efficacy by reviewing five observational studies, including two controlled clinical trials (Risch, 2020a). This study received strong scientific criticism and raised serious concern for openly promoting HCQ without strong clinical trial evidence (Fleury, 2020). The author has published a follow-up to that study that described seven additional studies in support of his earlier findings (Risch, 2020b). However, the data used in the study were either unpublished or without data. Further, none of the studies presented in the article was found to be large randomized controlled trials.

**FIGURE 2:**
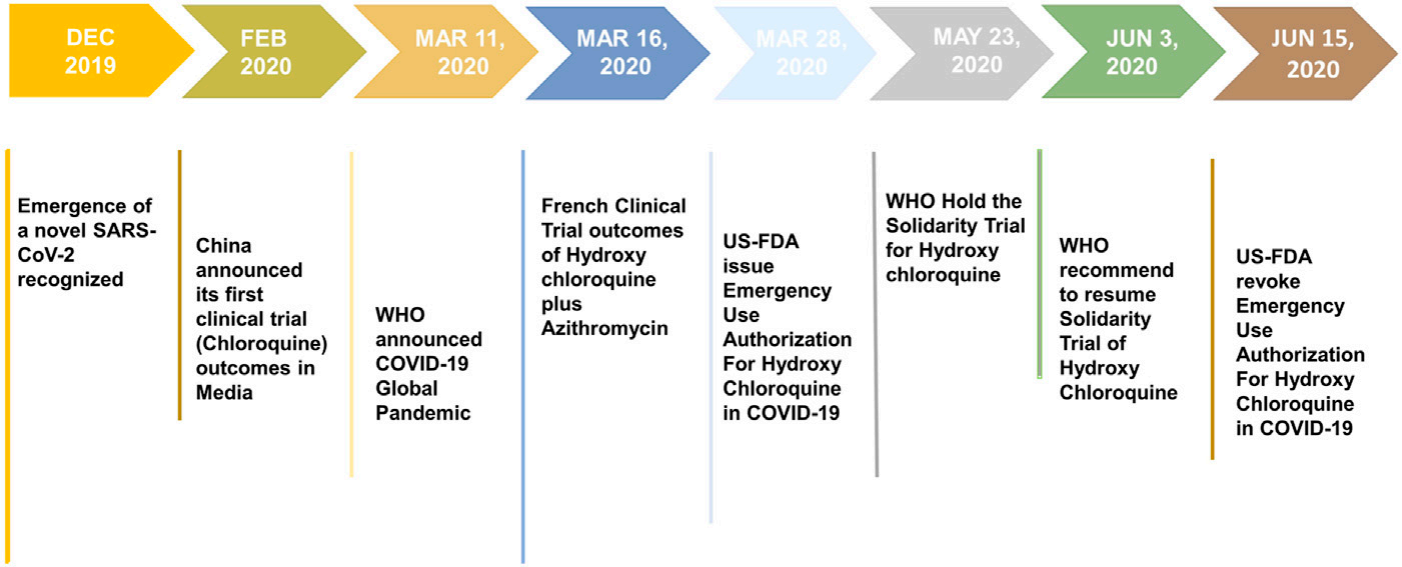
Various stages of development of chloroquine (CQ) and hydroxychloroquine (HCQ) as compassionate therapy.

The WHO launched a huge international clinical trial called “Solidarity” to assess the effectiveness and safety of certain drugs that could be rapidly deployed in the battle against COVID-19. CQ and HCQ are enlisted in the Solidarity trial along with four other antiviral drugs. A research group studied the use of hydroxychloroquine or chloroquine with or without azithromycin for the treatment of COVID-19 by critically analyzing an enormously large COVID-19 clinical data (96,032 patients) obtained from 671 hospitals (Mehra et al., 2020a). The retrospective analysis of these data did not confirm any potential benefit for in-hospital patients, and further reported that the drugs decreased the survival of COVID-19 cases and resulted in an increased risk of ventricular arrhythmias. The same research group published a similar research that did not confirm the potential risk of angiotensin-converting enzyme (ACE) inhibitor or angiotensin II receptor blockers (ARBs) for in-hospital COVID-19 patients (Mehra et al., 2020b). These findings greatly influenced the WHO to take major decision over ongoing clinical trials of CQ and HCQ. On May 23, 2020, the executive body of the “Solidarity trial” decided to put a temporary hold on the HCQ trial, because of some safety concerns. Shortly after the retraction of the studies that rattled the scientific community (Mehra et al., 2020a; Mehra et al., 2020b), on June 3, 2020, the executive group received a recommendation based on the mortality data and endorsed the continuation of the HCQ Solidarity trial. The stagewise development of CQ and HCQ in COVID-19 is summarized in Figure 2. Till March 29, 360 clinical trials are registered to evaluate the therapeutic efficacy and prophylactic action of both CQ (88) and/or HCQ (272) in COVID-19 patients (Clinical trials report table 2021, WHO). However, only a few of the researchers published preliminary results, while other studies are under process. Although existing clinical trial data support some beneficial effects of CQ and HCQ in COVID-19, some of the ongoing trials were canceled or stopped due to possible adverse effects. Thus far, clinical trial results obtained for HCQ from different studies majorly suffer due to limitations of small sample sizes. Neither the French nor Chinese studies conducted for CQ and HCQ were randomized clinical trials. The clinical trial investigators of University Hospital Institute Méditerranée Infection acknowledged the “small sample size” and also the side effects of HCQ. The results of a RECOVERY trial (Randomized Evaluation of COVID-19 therapy) were carried out in the United Kingdom; a large clinical trial aimed to identify potential treatments for hospitalized COVID-19 cases that did not support the use of HCQ (The Recovery Collaboration group, 2020).

The global pandemic of SARS-CoV-2 infection has spread out of control in several countries and caused considerable morbidity and mortality (Elissa et al., 2020). Thus, there is an urgent need for an effective treatment to cure COVID-19 patients and also to prevent community transmission. Overall, the antiviral activities of CQ and HCQ against several viral diseases, including novel coronaviruses, low costs, good safety profile, and preexisting supply chain, pave the path for entry of these drugs into the treatment guideline of COVID-19. CQ and HCQ have been currently authorized by many countries for treating COVID-19 on a compassionate basis with caution. On March 28, 2020, the U.S. Food and Drug Administration (FDA) has issued the EUA for the inclusion of HCQ in the treatment of COVID-19. Subsequently, on June 15, 2020, the U.S. FDA revoked the emergency use authorization (EUA) based on its ongoing analysis. The U.S. FDA further stated that both of these drugs show no benefit on mortality or in speeding recovery, and hence are unlikely to be effective in treating COVID-19 patients. Recently, on June 17, 2020, the WHO also announced to stop the Solidarity trial of HCQ in COVID-19. However, the WHO decided not to prohibit the use or evaluation of hydroxychloroquine in pre- or postexposure prophylaxis in COVID-19 patients.

## Conclusion

The worldwide spread of SARS-CoV-2 infection made the global healthcare system to confront an entirely new and unprecedented situation. Clinicians worldwide employed a drug repurposing strategy to find drugs that can stem the progression of this highly contagious disease. A plethora of literature evidence on the antiviral potential of CQ and HCQ against several types of viruses including coronaviruses and preliminary clinical data on therapeutic benefits observed with CQ and HCQ treatment in COVID-19 patients led to the FDA authorization of both CQ and HCQ for compassionate use against COVID-19. Furthermore, clinical trial reports from China and France speculated the claims on the anti–SARS-COV-2 efficacy of HCQ either alone or in combination with other drugs like azithromycin. Although preliminary reports supported the use of the antimalarial agents such as CQ and HCQ to treat this rampant COVID-19, subsequent hospital observations and evidence from the large randomized clinical trials of HCQ did not demonstrate any clinical benefits. Ultimately, HCQ as COVID-19 therapy has come to an end on June 15, 2020, as the U.S. FDA revoked the EUA authorization. However, the global search for an effective drug or vaccine remains continues inspiring hope in the battle against COVID-19. Furthermore, our study emphasizes the need for evidence-based treatment approaches from large randomized clinical trials to confront the ongoing COVID-19 pandemic and not the mere observational study that mislead the public healthcare system, which paralyzes the entire world.
